# Market Exclusivity of the Originator Drugs in South Korea: A Retrospective Cohort Study

**DOI:** 10.3389/fpubh.2021.654952

**Published:** 2021-04-06

**Authors:** Kyung-Bok Son

**Affiliations:** School of Pharmacy, Sungkyunkwan University, Seoul, South Korea

**Keywords:** pharmaceutical expenditure, the first generic, market competition, market exclusivity, South Korea

## Abstract

**Introduction:** Generic entry is a well-known driver of competition and cost containment.

**Objectives:** We aim to measure the market exclusivity of originator drugs and to determine what influences the entry of generics in South Korea.

**Methods:** A list of originator drugs approved by the authority from 2000 to 2013 and their corresponding generics were paired. An event history model was applied for a statistical estimation for the duration until generic entry and to identify abbreviating or prolonging factors on the duration.

**Results:** A total of 2,061 pairs of originator and generics were identified. The market exclusivity for the originator drugs, including NDAs and non-NDAs, has not notably changed. However, competition among non-NDAs was less common than we expected. We found delayed time to entry of generics in the long run, particularly for non-NDAs in injection forms and biologics, and this finding is partially associated with market attractiveness.

**Conclusion:** The authority should address the delayed availability of certain types of generic drugs. The government could provide information on off-patent pharmaceuticals with no generic competition, designate their corresponding submissions as prioritized in the review process, and provide additional market exclusivity when entering the market via a long period of exclusivity.

## Introduction

Generic entry is a well-known driver of competition and cost containment in the pharmaceutical sector (1). Authorities approve generics that present pharmaceutical equivalence and bioequivalence on the basis of comparisons with the originator drug (2, 3). Because generic manufacturers do not have to conduct direct research, they develop generics at a lower price than that of the originator drug (4). Given the bioequivalence and lower price, economic theory suggests that generics are perfect substitutions of the originator drugs to a rational consumer in the market (5).

The entry of a generic drug will trigger competition and significantly alter the market structure (6). The entry of a generic drug will end the monopoly rent enjoyed by originator manufacturers and transform the monopoly market to an oligopoly where the originator drug and generic drugs compete (7). Thus, the duration of market exclusivity of the originator drug and the timing of the entry of generic drugs are interesting topics from the perspective of research and policy.

Previous health economics or health policy literature has emphasized the consequences of generic entrants. The literature has focused on the effect of a generic entrant from the perspectives of the price of the originator drug and generics (8–11), switching behavior by physicians (12, 13), market share of generics (14, 15), and expansion of the market within a substance category (16). Paradoxically, research has not concentrated on the market exclusivity of the originator drug and what influences the entry of generics.

However, the timing of the entry of generics and factors affecting the entry of generics are important factors in the management of pharmaceutical expenditures (17–19). This study aims to measure the market exclusivity of originator drugs and to determine what influences the entry of generics in the South Korean market. To this end, we investigated the market exclusivity of the originator drugs; determined what influences the entry of generics; and suggested policy options to rationalize pharmaceutical expenditure in South Korea.

## Materials and Methods

This study investigates the market exclusivity of the originator drugs and the timing of the first generic entry. We defined an originator drug as a pharmaceutical that was the first to be granted marketing authorization, whereas the first generic was defined as the second pharmaceutical that was granted marketing authorization after the originator and has the same active ingredients, strength, and route of administration as the originator. A list of originator drugs approved by the Ministry of Food and Drug Safety (MFDS) from 2000 to 2013 and their corresponding generics were paired with baseline information. South Korea provides 6 years of data exclusivity for new drugs (20). Thus, we excluded originator drugs approved after 2014.

### Data Sources

We used two datasets provided by the Health Insurance Review and Assessment Services (HIRA) and the MFDS. First, the list of reimbursed medicines under the National Health Insurance Service (NHIS) was retrieved from the website of the HIRA. The list provides the characteristics of the pharmaceutical: generic and proprietary name of the pharmaceutical and its strength, manufacturer, and reimbursement price. Second, we extracted information on all pharmaceutical approved by the MFDS from 2000 to 2013. In particular, the Korea Pharmaceutical Information Service (KPIS) provides the similar characteristics of the pharmaceutical: generic and proprietary name of the pharmaceutical and its strength, anatomical therapeutic chemical (ATC) classification, substance type (including chemicals and biologics), manufacturer, and date of marketing approval. Using information on the generic name of the pharmaceutical and its strength, two datasets were merged.

### Variables

We are mainly interested in market exclusivity of the originator drugs. Market exclusivity was measured as the year difference between the date of regulatory approval of the originator drug and that of the corresponding first generic.

We choose a set of variables to understand variations in market exclusivity of originator drugs: the characteristics of the originator drug, the manufacturer, and the market. First, we categorized originator drugs into New Drug Application (NDA) and non-NDA. An NDA refers to “a drug of new materials, a substance with a chemical structure or construction that is wholly new, or a combination drug containing new materials as effective ingredients” in South Korea (21). Second, we categorized the characteristics of the originator drug based on the pharmaceutical's ATC classification, route of administration, substance type, and year of marketing authorization. Based on the number and characteristics of identified pharmaceuticals, ATC classification was categorized into four groups: alimentary tract and metabolism/blood and blood forming organs/cardiovascular system (A/B/C), antiinfectives for systemic use/antineoplastic and immunomodulating agents (J/L), musculo-skeletal system/nervous system (M/N), and others. Pharmaceuticals belong to A/B/C group indicate medicines prescribed for chronic diseases, including hypertension and diabetes, while pharmaceuticals belong to J/L groups include cancer drugs. The year of marketing authorization was grouped into three periods to note time trends: Period I (2000–04), Period II (2005–09), and Period III (2010–13). Third, we grouped the manufacturers of the pharmaceuticals into domestic and overseas. The Ministry of Trade, Industry and Energy provides the dataset to identify the origin of the manufacturers. Finally, we identified the characteristics of the market based on reimbursement price. Price was categorized into four groups: low-price (<1,000 KRW, ~0.86 USD), medium-price (between 1,000 and 10,000 KRW, approximately between 0.86 and 8.6 USD), high-price (between 10,000 and 100,000 KRW, approximately between 8.6 and 86 USD), and very-high-price (>100,000 KRW, ~86 USD) medicines.

### Statistical Analysis

We used two statistical analyses to understand market exclusivity of the originator. First, we used descriptive analyses to present the difference in market exclusivity between three periods, namely, period I (2000–04), period II (2005–09), and period III (2010–13). Second, we applied an event history model for a statistical estimation. The model, which is also known as a duration model, estimates the duration until an event (or generic entry) and identifies abbreviating or prolonging factors on the duration. As a univariate tool, we applied Kaplan-Meier survival estimates and conducted log-rank test to compare the generic entrance distributions of the samples. We applied the proportional hazard model as a multivariate tool. We presented two types of proportional hazard model: the simple and expanded. In the simple model, we included the characteristics of the originator drug, such as ATC classification, route of administration, substance type, and year of marketing authorization. We added characteristics of the manufacturer and market in the expanded model. Furthermore, we separated the pharmaceuticals into NDAs and non-NDAs in sub-group analyses. Data management and analysis were performed using R statistical software (version 3.4.3). Statistical significance is noted by *p*-values < 0.05.

## Results

### Subjects of the Study

Table 1 presents the characteristics of the subjects. During a 14-year period, a total of 2,061 pairs of originator and first generics were identified as the subjects. We categorized the subjects into three periods, namely, period I, period II, and period III, based on the approval year of the originator. Approximately, 40% (818 pairs), 37% (762 pairs), and 23% (481 pairs) of the subjects belong to periods I, II, and III, respectively. The proportion of oral forms in each period has increased, whereas that of injection and other forms has decreased. Similarly, the proportion of high- or very-high-priced pharmaceuticals has increased from 31% in period I to 41% in period III.

**Table 1:** Characteristics of the originator drugs.

**Variables**	**All** ** (*****n*** **=** **2,061)**		**Period I** ** (*****n*** **=** **818)**		**Period II** ** (*****n*** **=** **762)**		**Period III** ** (*****n*** **=** **481)**		***P*-value**
**The ATC classification**									
J/L	351	17%	127	16%	133	17%	91	19%	0.0595
A/B/C	677	33%	250	31%	255	33%	172	36%	
M/N	363	18%	143	17%	140	18%	80	17%	
Others	670	33%	298	36%	234	31%	138	29%	
**Route of administration**									
Oral	1,035	50%	360	44%	401	53%	274	57%	<0.0001
Injection	326	34%	153	37%	120	32%	53	32%	
Others	700	16%	305	19%	241	16%	154	11%	
**Type of substance**									
Chemicals	1,911	7%	771	6%	693	9%	447	7%	0.0398
Biologics	150	93%	47	94%	69	91%	34	93%	
**Manufacturers**									
Domestic	1,181	57%	527	64%	398	52%	256	53%	<0.0001
Overseas	880	43%	291	36%	364	48%	225	47%	
**New drug application**									
Yes	1,733	16%	694	15%	628	8%	411	15%	0.2719
No	328	84%	124	85%	134	82%	70	85%	
**Reimbursed price**									
Low	814	39%	347	42%	290	38%	177	37%	<0.0001
Medium	551	27%	216	26%	227	30%	108	22%	
High	483	23%	194	24%	169	22%	120	25%	
Very high	213	10%	61	7%	76	10%	76	16%	

*A, alimentary tract and metabolism; B, blood and blood forming organs; C, cardiovascular system; J, antiinfectives for systemic use; L, antineoplastic and immunomodulating agents; M, musculo-skeletal system; N, nervous system; Period I (2000–04), Period II (2005–09), Period III (2010–13)*.

### Market Exclusivity

Table 2 provides the market exclusivity of the originator drugs. However, our observations are right-censored, indicating that some of the originator drugs might experience generic competition over time. Thus, we separated the subject into the ongoing exclusivity group and the terminated exclusivity group and presented their market exclusivity. Terminated exclusivity indicates that generic drugs were granted marketing authorization, while ongoing exclusivity indicates that the originator drug constitutes a monopoly market without generic competition. Approximately, 45, 40, and 26% of originator drugs were grouped in the terminated group in periods I, II, and III, respectively. For pharmaceuticals belonging to the terminated group, the median of the exclusivity was 4.74, 4.32, and 2.00 years for periods I, II, and III, respectively. Similarly, the median values of the exclusivity for pharmaceuticals belonging to the ongoing group were 17.03, 12.29, and 7.34 years for periods I, II, and III, respectively.

**Table 2:** Market exclusivity of the originator drugs, including NDAs and non-NDAs.

	**All (*****n*** **=** **2,061)**						**NDAs (*****n*** **=** **328)**						**Non-NDAs (1,733)**					
	**Period I**		**Period II**		**Period III**		**Period I**		**Period II**		**Period III**		**Period I**		**Period II**		**Period III**	
	818		762		481		124		134		70		694		628		411	
**Exclusivity status**																		
Terminate	368	45%	306	40%	127	26%	73	59%	63	49%	7	10%	295	43%	243	39%	120	29%
Ongoing	450	55%	456	60%	354	74%	51	41%	71	41%	63	90%	399	57%	385	61%	291	71%
**Terminate**																		
Mean	5.59		4.37		2.66		9.18		6.62		6.51		4.71		3.78		2.44	
Median	4.74		4.32		2.00		8.19		6.71		6.87		3.49		2.51		1.91	
SD	4.77		3.58		2.44		3.45		2.89		0.73		4.64		3.51		2.32	
**Ongoing**																		
Mean	17.12		12.27		7.49		17.46		12.10		7.68		17.07		12.30		7.45	
Median	17.07		12.29		7.34		17.64		12.02		7.88		17.03		12.33		7.34	
SD	1.49		1.27		1.18		1.34		1.11		1.16		1.51		1.30		1.19	

*NDA, New Drug Application; Period I (2000–04), Period II (2005–09), Period III (2010–13)*.

We separated originator drugs into NDAs and non-NDAs and calculated their market exclusivity. Approximately, 59, 49, and 10% of the NDAs in periods I, II, and III were grouped in the terminated exclusivity group, respectively; the median of market exclusivity of NDAs in these periods was 8.19, 6.71, and 6.87 years, respectively. Note that South Korea provides 6 years of data exclusivity for NDAs. Thus, the median of market exclusivity for NDAs is longer than 6 years. Similarly, we calculated the market exclusivity for non-NDAs in periods I, II, and III. Approximately, 43, 39, and 29% of the non-NDAs in periods I, II, and III were grouped in the terminated exclusivity group, respectively. The median of market exclusivity of non-NDAs in these periods was 3.49, 2.51, and 1.91 years, respectively.

### Statistical Analysis

#### Kaplan-Meier Survival Analysis

Appendices 1–3 provide a descriptive overview of the difference in durations, including all pharmaceuticals, NDAs, and non-NDAs, using Kaplan-Meier estimates. The estimates present the conditional probability that generic will enter the market after a given period. In particular, the various curves in Appendix 1 indicate the probability that the originator drugs that will face generic competition after a specific year. The first graph in Appendix 1 presents a curve without group comparison. The remaining graphs present curves with group comparison, including period, substance type, presence of manufacturers in South Korea, ATC classification, route of administration, reimbursed price, and designation of NDAs. Because South Korea provides 6 years of data exclusivity for new drugs, the last curve in Appendix 1 for NDAs presents a plateau until 6 years after the marketing date of the originator drug. Additionally, the curve for NDAs went down steeply after 6 years, while the curve for non-NDAs went down smoothly during the study period. In the log-rank test, significant difference in generic entrance curves was observed in variables of substance type, ATC classification, route of administration, and reimbursed price.

Given the 6-year data exclusivity period granted to NDAs, we separated the subjects into NDAs and non-NDAs. Similar to Appendices 1–3 present curves for the probability of the originator drugs that will face generic competition after a specific year. Curves with group comparisons based on substance type, including chemicals and biologics, were similar for NDAs and non-NDAs. However, other remaining curves with group comparisons were different. For instance, curves with group comparisons of route of administration were different. More specifically, the conditional probability that a generic entry will occur exhibited the order of oral, injection, and other after 15 years for NDAs. However, the same probability exhibited the order of oral, others, and injection for non-NDAs. Significant difference in generic entrance curves was observed in variables of substance type, ATC classification, route of administration, and reimbursed price in Appendices 2, 3. Furthermore, the variable on period presented a significant difference in Appendix 2.

#### The Proportional Hazard Model

Table 3 provides results for the effects from the simple proportional hazard model. We fitted the simple model with four discrete factors: ATC classification, route of administration, substance type, and period based on the marketing approval year. Note that a positive coefficient indicates a short time to generic entry (timely generic competition), while a negative coefficient indicates a long time to generic entry (delayed generic competition). Thus, the time to generic entry for pharmaceuticals in injection form was delayed compared to that in oral form. Similarly, the time to generic entry for biologics was delayed compared to that for chemicals. However, the period variable was not significantly delayed or accelerated for the time to generic entry. Additionally, we separated the subjects into NDAs and non-NDAs and conducted the same analysis. Interestingly, we found that the time to generic entry for pharmaceuticals in injection form and biologics was delayed only for non-NDAs. Consistent with this result, the time to generic entry for pharmaceuticals approved in period III was delayed for NDAs.

**Table 3:** Results for the effects from the proportional hazard assumptions in the simple model.

	**All (*****n*** **=** **2,061)**			**NDAs (*****n*** **=** **328)**			**Non-NDAs (1,733)**		
	**Coefficient**	**Standard error**	***P*-value**	**Coefficient**	**Standard error**	***P*-value**	**Coefficient**	**Standard error**	***P*-value**
**The ATC classification (Reference J/L)**									
A/B/C	−0.2054	0.1075	0.0560	−0.3685	0.2363	0.1188	−0.2394	0.1256	0.0567
M/N	0.1776	0.1128	0.1153	0.5305	0.2350	0.0240	0.0606	0.1337	0.6501
Others	−0.3005	0.1190	0.0116	−0.1923	0.2565	0.4534	−0.3638	0.1379	0.0083
**Route of administration (Reference Oral)**									
Injection	−0.6440	0.0932	<0.0001	−0.2235	0.2189	0.3072	−0.7252	0.1037	<0.0001
Others	−0.1745	0.1180	0.1392	−1.2549	0.5417	0.0205	−0.1154	0.1239	0.3515
**Type of substance (Reference Chemicals)**									
Biologics	−0.5000	0.2027	0.0137	−0.5113	0.4278	0.2320	−0.5337	0.2316	0.0212
**Period (Reference Period I)**									
Period II	−0.0277	0.0794	0.7271	0.2057	0.1928	0.2861	−0.0575	0.0886	0.5160
Period III	−0.1938	0.1070	0.0702	−0.9205	0.4088	0.0244	−0.1550	0.1125	0.1682

*NDA, New Drug Application; Period I (2000–04), Period II (2005–09), Period III (2010–13)*.

Two variables of manufacturer and reimbursed price were added in the expanded model in Table 4. The expanded model produced results that were consistent with those of the simple model. Additionally, we found that the time to generic entry for medium-, high-, and very-high-price pharmaceuticals was delayed compared to that for low-price pharmaceuticals. We also found that the time to generic entry for pharmaceuticals produced by overseas manufacturers was accelerated compared to that for pharmaceuticals produced by domestic manufacturers for NDAs.

**Table 4:** Results for the effects from the proportional hazard assumptions in the expanded model.

	**All (*****n*** **=** **2,061)**			**NDAs (*****n*** **=** **328)**			**Non-NDAs (1,733)**		
	**Coefficient**	**Standard error**	***P*-value**	**Coefficient**	**Standard error**	***P*-value**	**Coefficient**	**Standard error**	***P*-value**
**The ATC classification (Reference J/L)**									
A/B/C	−0.3440	0.1140	0.0025	−0.6725	0.2865	0.0189	−0.3305	0.1293	0.0105
M/N	0.04252	0.1178	0.7183	0.2646	0.2665	0.3206	−0.0157	0.1364	0.9080
Others	−0.3980	0.1229	0.0012	−0.4011	0.2748	0.1443	−0.4322	0.1406	0.0021
**Route of administration (Reference Oral)**									
Injection	−0.3729	0.1166	0.0013	0.2721	0.2840	0.3380	−0.4799	0.1329	0.0003
Others	−0.0113	0.1258	0.9280	−0.9886	0.5601	0.0775	−0.4785	0.1340	0.7618
**Type of substance (Reference Chemicals)**									
Biologics	−0.4690	0.2109	0.0261	−0.7183	0.4489	0.1095	−0.4799	0.2409	0.0464
**Period (Reference Period I)**									
Period II	0.0062	0.0802	0.9380	0.2439	0.1968	0.2150	−0.0202	0.0897	0.8218
Period III	−0.1305	0.1086	0.2296	−0.7988	0.4165	0.0551	−0.0958	0.1143	0.4015
**Manufacturers (Reference Domestic)**									
Overseas	0.0282	0.0767	0.7126	0.5818	0.2307	0.0116	−0.0396	0.0856	0.6431
**Reimbursed price (Reference Low)**									
Medium	−0.2435	0.0940	0.0103	−0.2739	0.2351	0.2440	−0.2438	0.1055	0.0209
High	−0.4867	0.1312	0.0002	−0.9269	0.3273	0.0046	−0.3899	0.1486	0.0086
Very high	−0.4792	0.1904	0.0118	−1.1419	0.4461	0.0104	−0.4181	0.2138	0.0505

*NDA, New Drug Application; Period I (2000–04), Period II (2005–09), Period III (2010–13)*.

## Discussion

Timely entry of generic drugs is a key driver of competition and cost containment in the pharmaceutical sector. Thus, understanding the timing of the entry of generics and factors affecting their entrance are essential to rationalize pharmaceutical expenditures. To this end, we measured the market exclusivity of the originator drugs and identified what influences the entry of generics in South Korea.

### Trends in Generic Entrants

According to our observations, the market exclusivity for the originator drugs has not notably changed. For instance, the period was not a significant factor in our proportional hazard model. This finding is consistent with previous literature. Son et al. (22) evaluated the effect of the patent linkage system on the patent challenge and market exclusivity of NDAs in South Korea. The authors calculated the effective market exclusivity for NDAs approved from 2007 to 2011 and concluded that the market exclusivity had not significantly changed after the introduction of the patent linkage system. Additionally, we updated similar results for non-NDAs in the current study.

However, it is noteworthy to compare NDAs and non-NDAs from the perspectives of market competition. In the Kaplan-Meier estimates, the curve for NDAs decreased steeply after 6 years from the marketing approval of the originator drug. However, the curve for non-NDAs smoothly decreased from the marketing approval of the originator drug. Interestingly, the conditional probability that the generic entry will occur after 15 years of the approval of originator drugs was higher for NDAs than for non-NDAs. Similarly, we found that the proportion of the terminated group was higher for NDAs than for non-NDAs in periods I and II. These observations indicate that competition among non-NDAs in the long run was less common than we expected.

### Factors Affecting Generic Entry

In the economic literature, it is well-documented that generic entry is driven by a variety of factors: manufacturer variables (23–25); pharmaceutical approval process in an authority (26–28); and markets attractiveness (29–34). First, manufacturer variables indicate the availability and cost of the raw materials, manufacturing processes and their corresponding cost as well as manufacturing and marketing experience with similar pharmaceutical products (23–25). Second, the pharmaceutical approval process in an authority includes quality of submissions that a manufacturer prepares and the review process that an authority provides (26–28). Finally, market attractiveness includes the size of the patient population being treated or projected profits in the market (31–33). More specifically, it was reported that the number of generic manufacturers is reduced for older orphan drugs (30), while the number of generic manufacturers entering a market is greater for pharmaceuticals with higher sales (29, 34). Furthermore, pricing and reimbursement policies of originator and/or generic drugs might influence in the timing of generic entrant (35).

Some of these factors are consistent in our study. We found that generic entries for biologics (reference chemicals) and pharmaceuticals in injection forms (reference oral forms) were delayed, indicating that manufacturer variables, including cost of the raw materials and manufacturing process, are critical factors in the entry of generics. However, interesting results were observed when we separated the subjects into NDAs and non-NDAs. Generic entries for biologics and pharmaceuticals in injection forms were delayed only for non-NDAs. The difference between NDAs and non-NDAs could be explained by their market attractiveness. During the 6-year period of data exclusivity given to NDAs, the market for the majority of NDAs continuously grew (36), indicating that projected profits for NDAs after 6 years of data exclusivity are higher than those of non-NDAs. In a similar vein, the market for NDAs manufactured by overseas manufacturers is larger than the market for NDAs manufactured by domestic manufacturers. Thus, the variables of overseas manufacturers (reference domestic manufacturers) in our proportional hazard model significantly accelerated generic entries in the market.

### Policy Implications Regarding Delayed Availability of Generic Drugs

In this study, we found delayed time to entry of generic drugs in the long run, particularly for non-NDAs in injection forms and biologics, and this finding partially associated with market attractiveness. In this context, the role of the MFDS to address the delayed availability of generic drugs could be revisited (31, 37).

As previously discussed, pharmaceutical approval process in the authority matters. Thus, an expedited (or prioritized) review process for the first generic applications or products with less than three competitors might be established in South Korea to attract (additional) manufacturers enter a market (38). In the same vein, the MFDS could provide information on off-patent pharmaceuticals with “no (or inadequate) generic competition” to manufacturers and designate their corresponding submissions as prioritized in the review process (39). However, the effects of these measures in the entry of generics are not clear and require additional empirical evidence and contexts for the market (31). For instance, the main reason of fewer generics in the United States is the limited demand for additional generic drugs and their lower potential profits. It is noteworthy that generic drug prices in South Korea are higher than that of other high-income countries, indicating that lower profits for generic drugs might not be applicable in South Korea. Finally, the additional first generic exclusivity for submissions for pharmaceuticals with “no (or inadequate) generic competition” could be devised. South Korea introduced a 9-month first generic exclusivity, which is one of compartments in the patent linkage system, on March 15, 2015 (40). In the patent linkage system, the first generic applicant who has challenged a patent and obtained a favorable decision could be granted a 9-month period of market exclusivity (22). Similarly, the first generic entering the market with a long period of exclusivity of originator drugs could be granted market exclusivity, and additional market exclusivity might encourage generic entrants in the market.

### Strengths and Limitations

We utilized two datasets provided by the MFDS and the HIRA and included all reimbursed pharmaceuticals in South Korea. Thus, the study findings might be generalizable to all types of pharmaceuticals. However, this study has limitations that are mainly attributed to data availability. We could not access information on the prescriptions and clinical effectiveness of the originator drug, indicating limited information on the characteristics of the originator drug and market. In particular, information on the number of prescriptions could be merged with the currently available dataset to fully understand the effect of generic entries from the perspectives of volume and/or value in the South Korean market. In a similar vein, we did not control for the effect of regulations in market exclusivity of originator drugs. Pricing and reimbursement policy could incentivize or dis-incentivize generic entrance.

## Conclusions

In this study, we investigated the market exclusivity of originator drugs, and identified factors affecting the timely availability of generic drugs in the South Korean market. Market exclusivity for originator drugs has not notably changed. However, it is noteworthy to compare market exclusivity of NDAs and non-NDAs from the perspective of competition. In particular, competition among non-NDAs in injection forms and biologics in the long run was less common than we expected. We suggested that the MFDS should address the delayed availability of certain generic drugs. The South Korean government could provide information on off-patent pharmaceuticals with no generic competition to manufacturers, designate their corresponding submissions as prioritized in the review process, and provide additional market exclusivity when entering the market via a long period of exclusivity.

## Data Availability Statement

The study analyzed data from publicly accessible datasets:https://www.hira.or.kr/bbsDummy.do?pgmid=HIRAA030014050000https://biz.kpis.or.kr/kpis_biz/index.jsp.

## Author Contributions

K-BS developed the concept the manuscript, undertook the analysis, and wrote the manuscript.

## Conflict of Interest

The author declares that the research was conducted in the absence of any commercial or financial relationships that could be construed as a potential conflict of interest.

## Supplementary Material

Appendix 1 Kaplan-Meier estimates for all medicines.

Appendix 2 Kaplan-Meier estimates for NDAs.

Appendix 3 Kaplan-Meier estimates for non-NDAs.
