# Supplementary figures and images for: Market Exclusivity of the Originator Drugs in South Korea: A Retrospective Cohort Study
Source: Front Public Health. 2021 Apr 6;9:654952. doi: 10.3389/fpubh.2021.654952 (PMC8056004; doi:10.3389/fpubh.2021.654952)

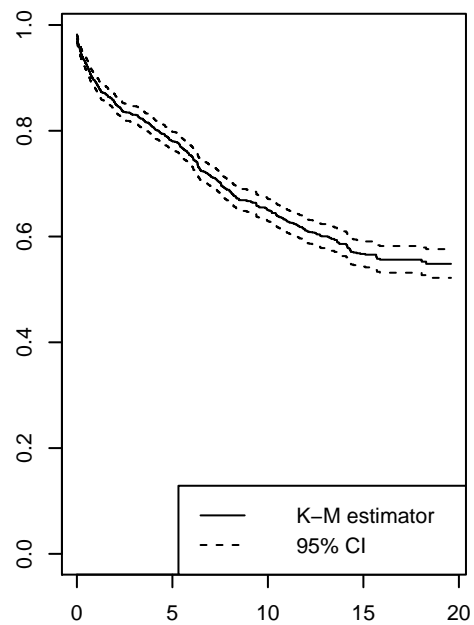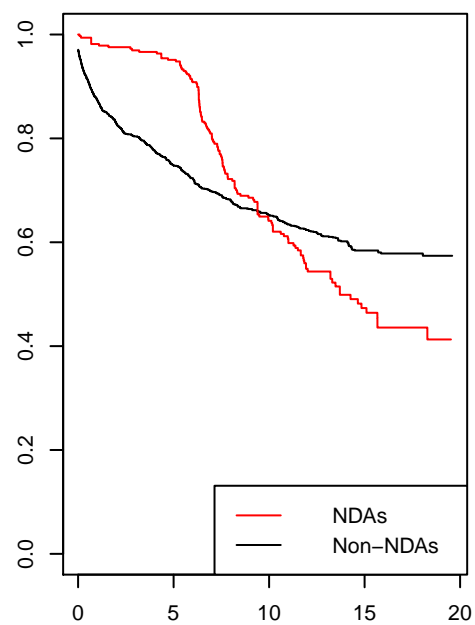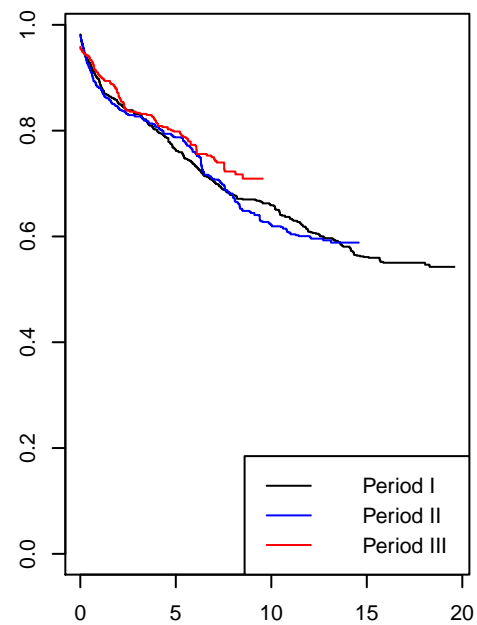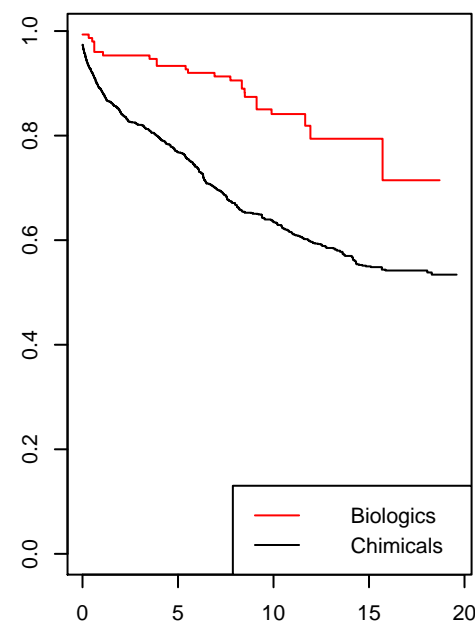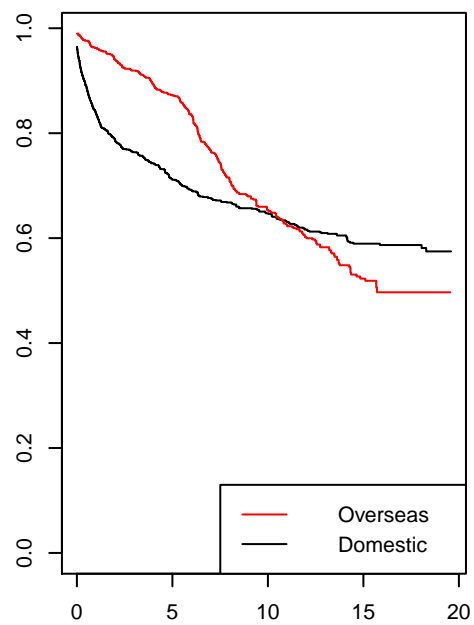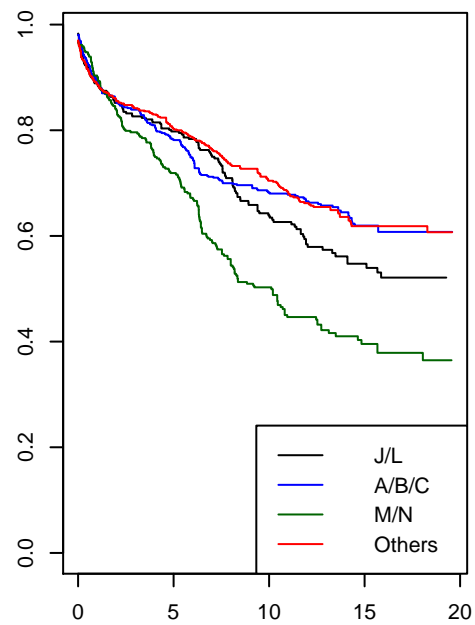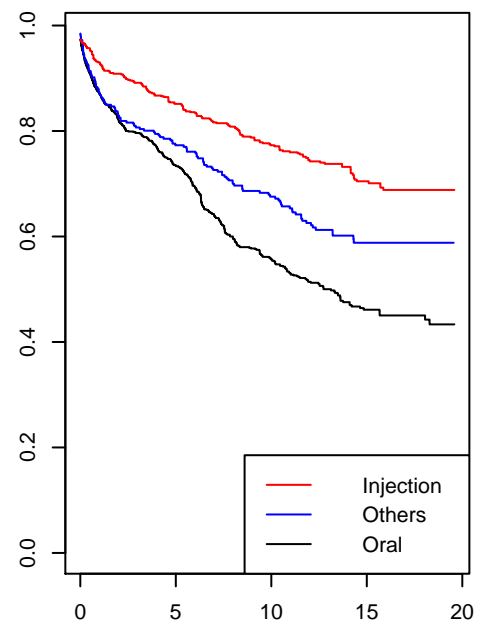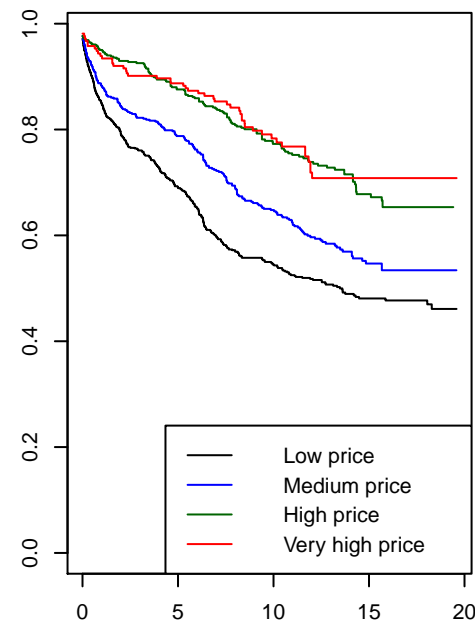

Supplement: Appendix 1 — Kaplan-Meier estimates for all medicines. [file Data_Sheet_1.PDF]

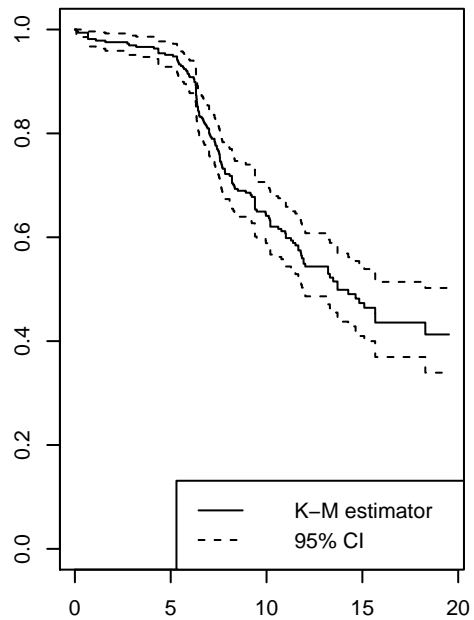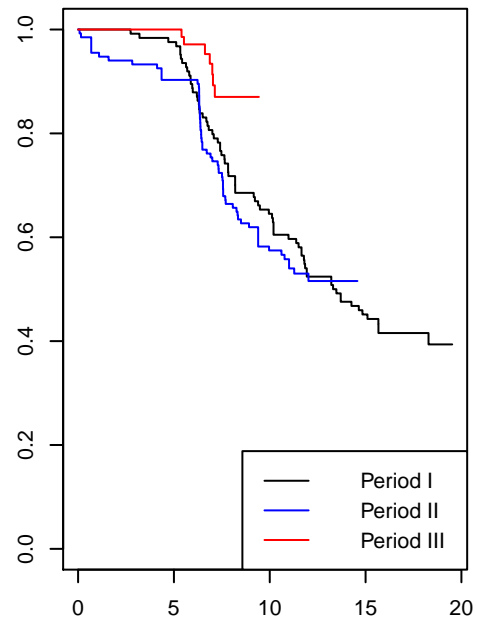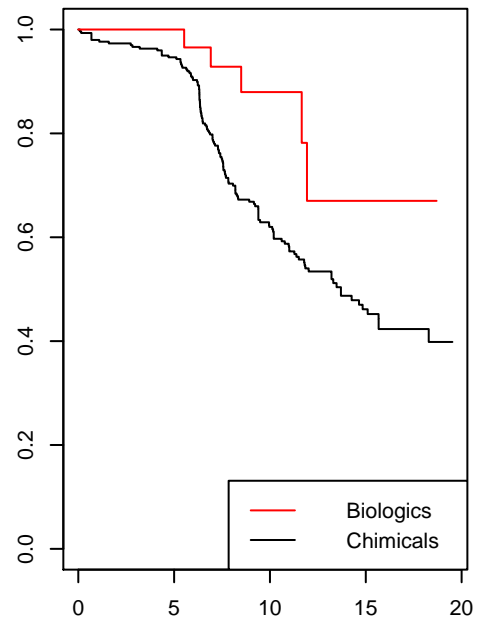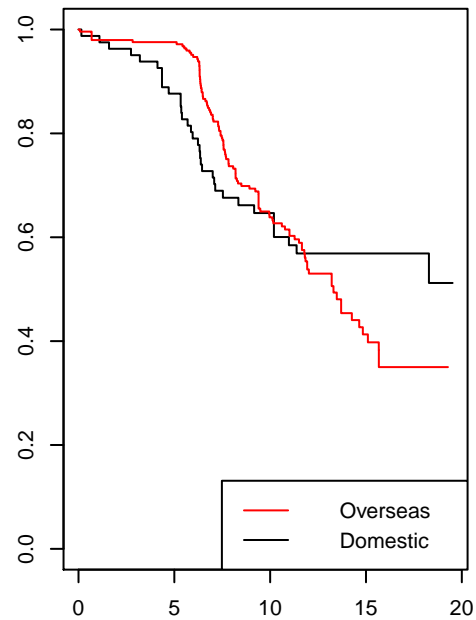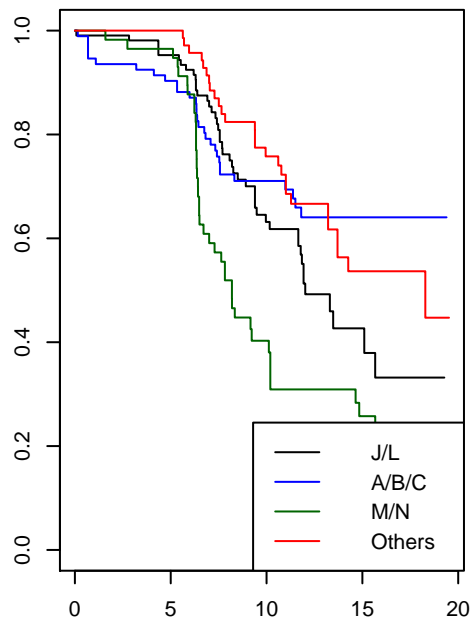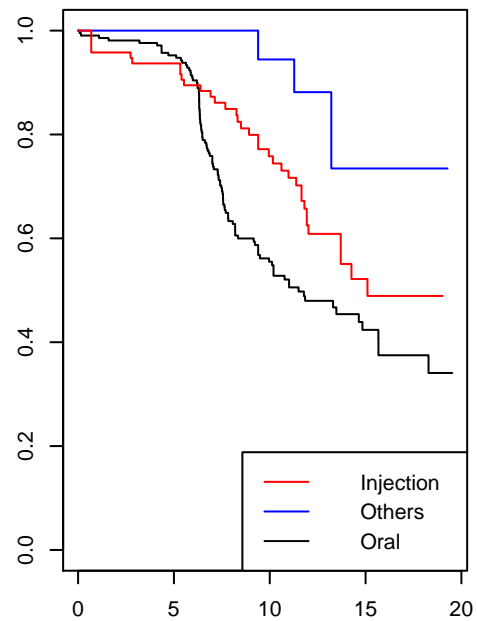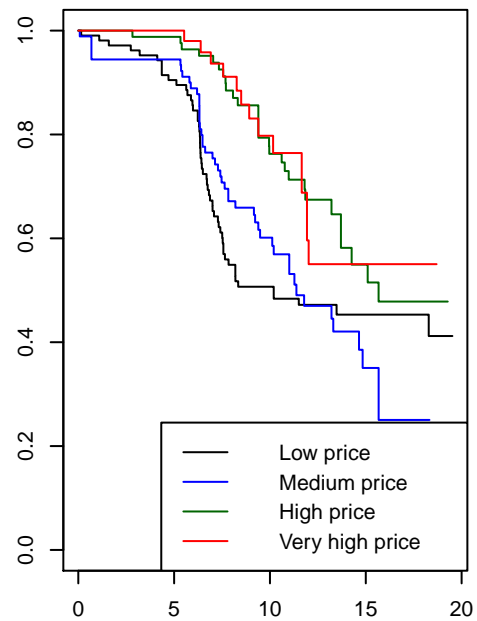

Supplement: Appendix 2 — Kaplan-Meier estimates for NDAs. [file Data_Sheet_2.PDF]

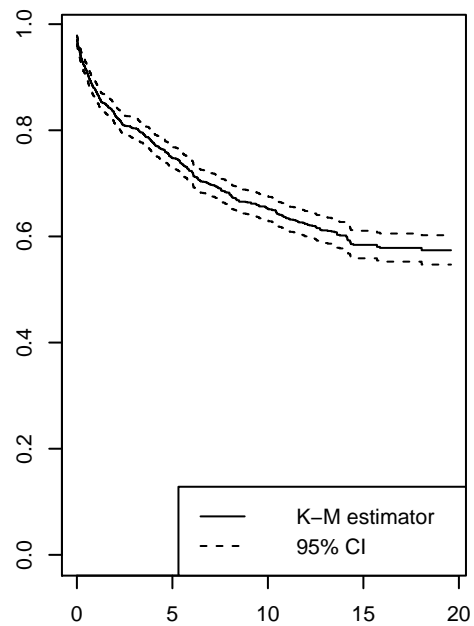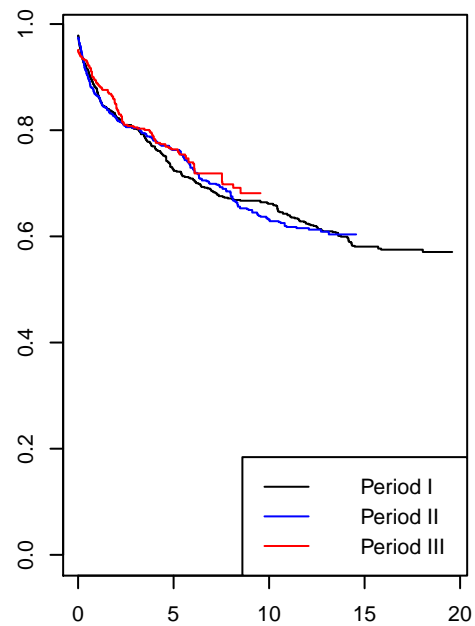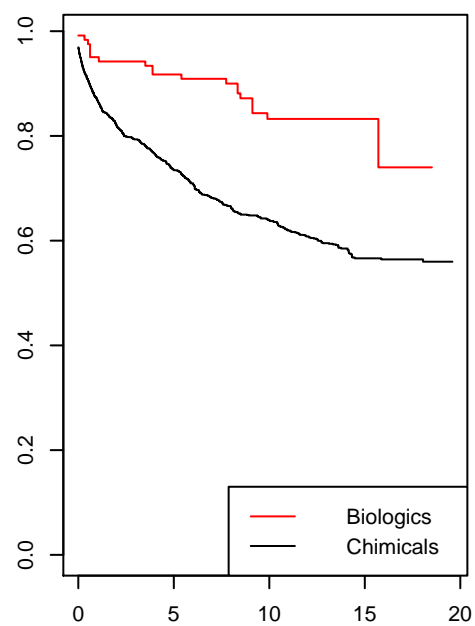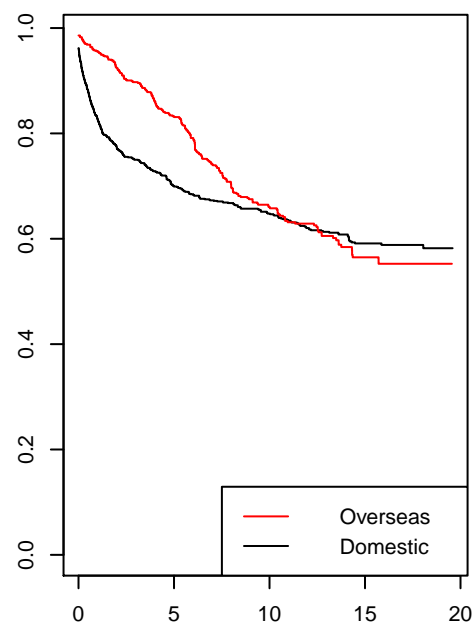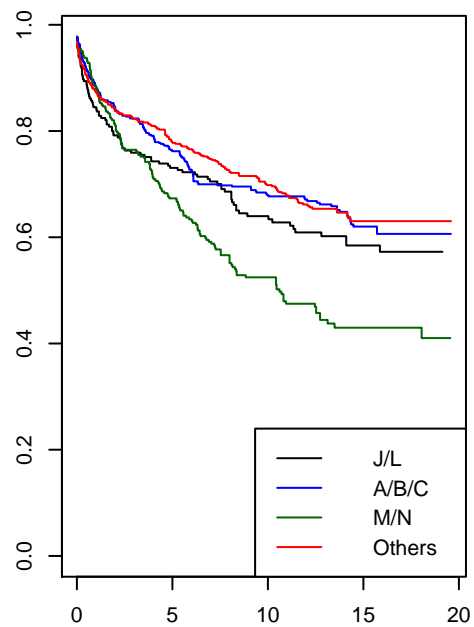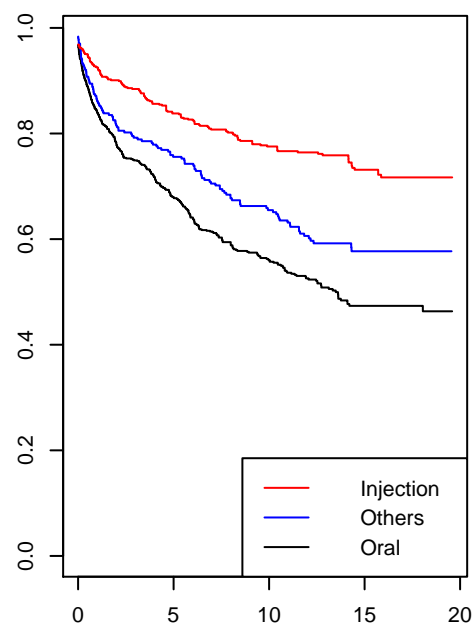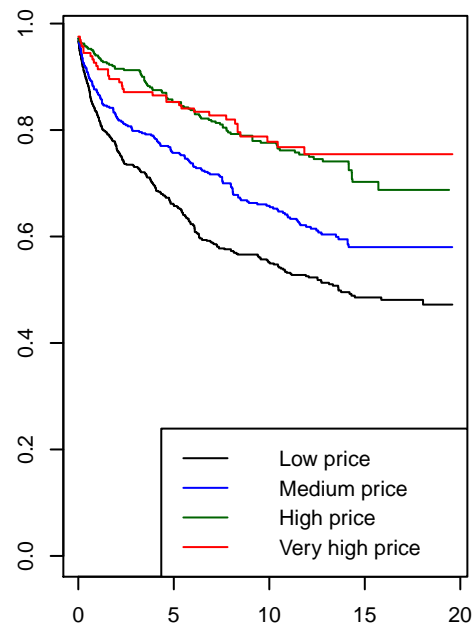

Supplement: Appendix 3 — Kaplan-Meier estimates for non-NDAs. [file Data_Sheet_3.PDF]
